# Comment on “Efficacy of Cosmetic Debridement and Suture With Recombinant Human EGF in Maxillofacial Trauma: A Meta‐Analysis”

**DOI:** 10.1111/jocd.70596

**Published:** 2025-12-09

**Authors:** Shyam Sundar Sah, Abhishek Kumbhalwar

**Affiliations:** ^1^ SR Sanjeevani Hospital Kalyanpur, Siraha Nepal; ^2^ Dr. D. Y. Patil Medical College Hospital and Research Centre Dr. D. Y. Patil Vidyapeeth (Deemed‐to‐be‐University) Pimpri, Pune Maharashtra India; ^3^ Dr. D. Y. Patil Dental College and Hospital Dr. D. Y. Patil Vidyapeeth (Deemed‐to‐be‐University) Pimpri, Pune Maharashtra India

**Keywords:** aesthetic wound healing, cosmetic debridement, maxillofacial trauma, recombinant human epidermal growth factor, scar assessment

Dear Editor,

We read with great interest the meta‐analysis by Yao and Yi, which synthesized six randomized controlled trials evaluating the combined use of cosmetic debridement and recombinant human epidermal growth factor (rhEGF) for maxillofacial trauma [1]. The authors are commended for integrating quantitative data on wound healing time, scar outcomes, and cytokine modulation, an area of growing importance in aesthetic and reconstructive facial surgery. Their inclusion of serum inflammatory biomarkers adds mechanistic depth beyond traditional scar scales. However, several aspects merit further discussion.

The literature base in this analysis is drawn exclusively from studies published in Chinese clinical databases, with all included trials showing moderate methodological quality based on the Newcastle‐Ottawa Scale. This concentration of data from a single regional context may introduce cultural and procedural homogeneity that limits generalizability. Aesthetic wound care is strongly influenced by population‐specific surgical techniques, skin phototypes, and scar phenotypes; thus, outcomes observed in homogeneous East Asian cohorts may not reflect scar dynamics in populations with higher Fitzpatrick skin types, where hypertrophic scarring risk differs significantly [2]. Broader inclusion of multinational evidence could refine external validity and guide protocol harmonization across global aesthetic practices.

While the authors reported favorable odds ratios and mean differences using both fixed‐ and random‐effects models, treatment of heterogeneity in cytokine outcomes raises interpretive challenges. Interleukin‐6 (IL‐6) and tumor necrosis factor‐alpha (TNF‐α) analyses exhibited high *I* ^2^ values (> 80%), yet conclusions were drawn regarding anti‐inflammatory efficacy. Given that cytokine levels fluctuate according to injury severity, timing of blood sampling, and concurrent antibiotic use, such heterogeneity may reflect more than biological variance [3]. Without standardizing postoperative sampling windows, pooled estimates risk overstating biochemical uniformity. Clinically, this uncertainty affects the reliability of rhEGF's anti‐inflammatory claim as a wound‐modulating adjunct.

Selection of outcome measures emphasizes short‐term re‐epithelialization and six‐month scar indices (Vancouver Scar Scale and Patient and Observer Scar Assessment Scale) but omits long‐term texture and pigmentation outcomes, which are decisive in aesthetic satisfaction. In facial trauma repair, late‐phase remodeling, including collagen alignment and melanocyte redistribution, determines lasting cosmetic results [4]. Future quantitative syntheses could integrate digital skin imaging or three‐dimensional scar morphometry to provide a more objective appraisal of late aesthetic recovery, bridging the gap between early wound metrics and patient‐perceived normalcy.

The discussion's focus on rhEGF's biochemical mechanisms may understate the procedural component of “cosmetic debridement” [5]. The finesse of tissue edge approximation, suture material selection, and tension distribution often exerts equal influence on scar quality as molecular adjuvants. Without stratifying for surgical technique standardization across included trials, the isolated contribution of rhEGF remains uncertain. Clinically, this distinction matters because optimizing technique may yield similar results at lower pharmacologic costs, especially in low‐resource settings where topical growth factors are unavailable.

In summary, while Yao and Yi present encouraging pooled evidence supporting rhEGF as a cost‐effective adjunct for maxillofacial wound care, interpretation of inflammatory modulation, aesthetic durability, and technique‐dependent variability warrant cautious application. Refinement through multicenter, multi‐ethnic randomized trials with standardized biomarker timing and digital scar analytics could better position rhEGF within evidence‐based cosmetic reconstructive algorithms.

## Author Contributions

Shyam Sundar Sah: Conceptualization, methodology, writing – original draft, writing – review and editing. Abhishek Kumbhalwar: Validation, supervision, project administration, writing – original draft, writing – review and editing.

## Funding

The authors have nothing to report.

## Disclosure

Generative AI tools, including Paperpal and ChatGPT 5, were utilized solely for language, grammar, and stylistic refinement. These tools had no role in the conceptualization, data analysis, interpretation of results, or substantive content development of this manuscript. All intellectual contributions, data analysis, and scientific interpretations remain the sole work of the authors. The final content was critically reviewed and edited to ensure accuracy and originality. The authors take full responsibility for the accuracy, originality, and integrity of the work presented.

## Ethics Statement

The authors have nothing to report.

## Consent

The authors have nothing to report.

## Conflicts of Interest

The authors declare no conflicts of interest.

## Data Availability

The authors have nothing to report.
